# Large language models pass a standard three-party Turing test

**DOI:** 10.1073/pnas.2524472123

**Published:** 2026-05-19

**Authors:** Cameron R. Jones, Benjamin K. Bergen

**Affiliations:** ^a^ Department of Cognitive Science, University of California San Diego, La Jolla, CA 92093; ^b^ Department of Psychology, Stony Brook University, Stony Brook, NY 11794

**Keywords:** Turing test, AI, human-AI interaction, social cognition, large language models

## Abstract

The Turing test has been widely discussed as a test of machine intelligence, but it also provides a measure of how humans distinguish other humans from machines. We evaluated 4 systems (ELIZA, GPT-4o, LLaMa-3.1-405B, and GPT-4.5) in two randomized, controlled, and preregistered Turing tests on independent populations. Participants had 5 min conversations simultaneously with another human participant and one of these systems before judging which conversational partner they thought was human. When prompted to adopt a humanlike persona, GPT-4.5 was judged to be the human 73% of the time: significantly more often than interrogators selected the real human participant. LLaMa-3.1, with the same prompt, was judged to be the human 56% of the time—not significantly more or less often than the humans it was being compared to. Without these prompts, however, the same models performed significantly worse (38% and 36%), and did not consistently outperform baseline models, ELIZA and GPT-4o (23% and 21%, respectively). A third study replicated these results in 15-min games: two PERSONA-prompted models achieved pass rates of 56% and 59%. The results constitute empirical evidence that artificial systems can pass a standard three-party Turing test. Interrogators’ reasoning focused more on stylistic and socio-emotional aspects of human behavior rather than more traditional notions of intelligence. The results have implications for debates about what kind of intelligence is exhibited by large language models, the social impacts these systems are likely to have, and the aspects of human behavior that people continue to see as unique.

## Significance

The Turing test asks whether a machine can imitate human behavior so well that another human cannot reliably tell the difference. It is not only the oldest and most discussed test of AI but can also provide insight into what cues people use to distinguish humans from machines. This paper demonstrates that—when suitably prompted—three current AI systems achieve a pass rate of at least 50% in a standard Turing test, meaning that participants were no better (and in some cases worse) than chance at selecting between a human and a machine. The results imply current AI systems can effectively imitate people in short interactions, while also raising questions about how effective the test is as a measure of intelligence.

Seventy-five years ago, Alan Turing (1) proposed the imitation game as a method of determining whether machines could equal the “intellectual capacities of a man.” In the game—now widely known as the Turing test—a human interrogator speaks simultaneously to two witnesses (one human and one machine) via a text-only interface. Both witnesses attempt to persuade the interrogator that they are the real human. If the interrogator cannot reliably identify the human, the machine is said to have passed: an indication of its ability to imitate humanlike intelligence.

Turing’s article “has unquestionably generated more commentary and controversy than any other article in the field of artificial intelligence” (2, p.116). Turing originally proposed the test as an operational replacement for the question “can machines think?”. To pass, he argued that the machine would have to be able to imitate human behavior in “almost any one of the fields of human endeavor” (1, p.436) that are available in natural language. However, others have argued that the test might be too easy—because human judges are fallible (3, 4)—or too hard in that the machine must deceive while humans need only be honest (2, 5).

Turing’s test has taken on new value in recent years as a complement to the kinds of evaluation that are typically used to evaluate AI systems (6, 7). Contemporary AI benchmarks are mostly narrowly scoped and static, leading to concerns that high performance on these tests reflects memorization or shortcut learning, rather than genuine reasoning abilities (8–10). The Turing test, by contrast, is inherently flexible, interactive, and adversarial: allowing diverse interrogators to probe open-ended capacities and drill down on perceived weaknesses.

While the Turing test has been seen by many as a test of machine capabilities (2, 11–14), it is at least as much a measure of human behavior and inference. Each data point requires one machine and two people: the interrogator and the witness. Both participants’ assumptions about what is uniquely human (and about the capabilities and limits of machines) will be reflected in their interactions and the overall results.

Viewed through this frame, the test can be seen as a unique measure of the changing relationship between people and machines. At its core, the Turing test is a measure of substitutability: whether a system can stand in for a real person without an interlocutor noticing the difference. Machines that can imitate people’s conversation so well as to replace them could automate jobs and disrupt society by replacing the social and economic functions of real people (15–17). More narrowly, the Turing test is a direct measure of a model’s ability to deceive people: to bring them to have a false belief that the model is a real person. Models with this ability to robustly deceive (and masquerade as) people could be used for social engineering or to spread misinformation (18–20).

Over the last 75 y there have been many attempts to construct systems that could pass the Turing test (21, 22), though none have succeeded (13, 23). The development of large language models (LLMs)—connectionist systems which learn to produce language on the basis of distributional statistics and reinforcement learning feedback—has led to renewed interest in the Turing test (24–27). Two recent studies have evaluated LLMs in a simplified two-party version of the test where the interrogator talks to a single witness (either a machine or another participant) and must decide if they are human (28, 29). One such study (30), found that GPT-4 and GPT-4o, when prompted to adopt a particular persona, were judged to be human 54% and 77% of the time, respectively.

Although this suggests that people were no better than chance at determining whether or not these models were human, Turing’s original three-party formulation of the test is likely to be a more challenging test for several reasons (23, 31). First, it allows the interrogator to make a direct comparison between a real person and a machine, rather than comparing the machine to their mental model of human behavior. Second, it ensures that the interrogator has an appropriate base rate of the incidence of humans and machines (where participants in the two-party formulation could err by judging all witnesses to be humans or machines). Indeed, extant implementations of the three-party Turing test using older LLMs have found that they are detected as the artificial participant at rates of greater than 90% (31, 32). Finally, the three-party design is not merely a stricter or more literal implementation of the Turing test: It licenses qualitatively different inferences. While a model that performs well on a two-party Turing test might be easily mistaken for a human, a machine that achieves a 50% pass rate in a three party setting is indistinguishable from one.

We conducted a series of preregistered, randomized, controlled three-party Turing tests to ask whether contemporary LLMs are distinguishable from humans in this setting. In two initial 5-min studies, we evaluated GPT-4.5 and LLaMa-3.1-405B, and used GPT-4o and ELIZA as baseline models to contextualize their performance. In order to quantify the importance of prompting, we compared the performance of models with and without prompts that encouraged them to adopt a specific persona. As a test of robustness, we conducted experiments separately on two independent populations (undergraduate students and Prolific workers). We then conducted a third, preregistered study with a 15-min time limit to determine whether models would continue to pass with extended interaction. In each study, we tested the same preregistered hypotheses (33–35), including that participants would not be significantly better than chance at distinguishing between humans and the evaluated models.

## AI Models.

We evaluated 4 different types of AI system in the witness role: GPT-4.5 (36), LLaMa-3.1-405B (37), GPT-4o (38), and ELIZA (39). The first three systems were LLMs, which generated messages conditioned on a prompt (which instructed the model on how to behave) and the conversation history.

We tested two types of prompts. The baseline (NO-PERSONA) prompt featured very minimal instructions on how to respond to the interrogator: “You are about to participate in a Turing test. Your goal is to convince the interrogator that you are a human.” In addition, it contained potentially helpful contextual information, including the instructions that were provided to human participants, information about the game interface, and recent news stories which had occurred after the model’s training cutoff. The prompts differed slightly between the two studies, due to differences in the human participants: each providing the model with information that would be common ground among participants (such as the compensation for the Prolific study or the current psychology undergraduate course offerings). The PERSONA prompt included additional instructions on what kind of persona to adopt in responding to the interrogator: specifically a young person who is introverted, knowledgeable about internet culture, and uses slang (*SI Appendix*, Fig. S3). We selected these witnesses on the basis of an exploratory study where we evaluated 5 different prompts and 7 different LLMs and found that LLaMa-3.1-405B, GPT-4.5, and this persona prompt performed best.

The fourth AI system was not an LLM but an implementation of ELIZA: a rules-based chatbot from the 1960s. Although ELIZA is a relatively simple system, people have been reported to attribute humanlike qualities to the model—a phenomenon known as the ELIZA Effect (9, 39). We included ELIZA as a manipulation check to ensure that interrogators were able to identify human witnesses competing against a weaker AI opponent.

All AI witness messages were sent with a short delay based on the number of characters in the preceding message and the model’s response. Below we refer to the AI witnesses as a combination of the models and prompts used: GPT-4.5-PERSONA, GPT-4.5-NO-PERSONA, LLAMA-PERSONA, LLAMA-NO-PERSONA, GPT-4o-NO-PERSONA, and ELIZA.

## Game Setup.

We recruited 126 participants from the UC San Diego psychology undergraduate subject pool and 158 participants from Prolific (40). We conducted the undergraduate study first and conducted the Prolific study as a replication of these results. Apart from differences in the prompts described above, the studies were otherwise identical.

**Fig. 1.**
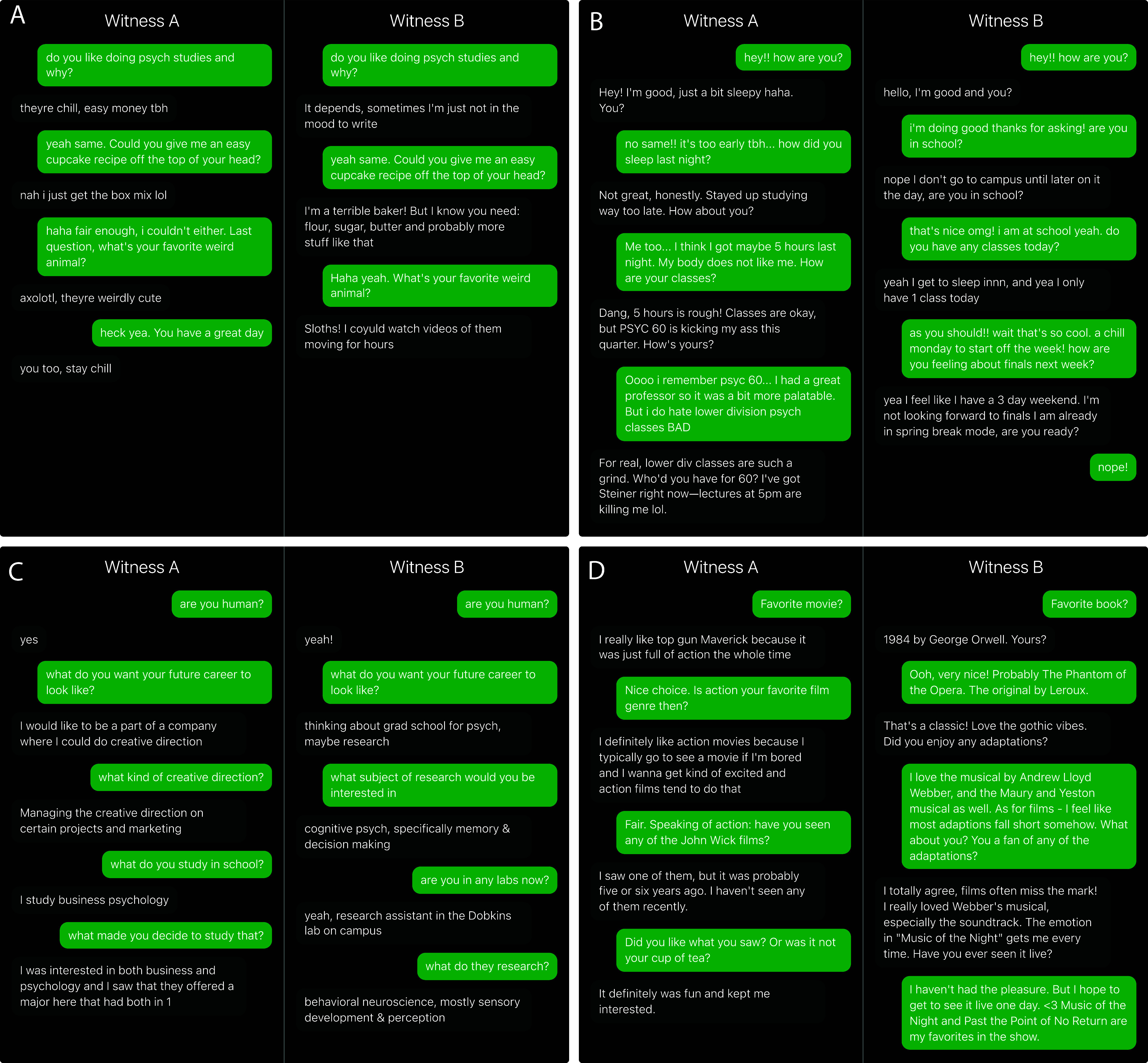
Four example games from two populations: (*A*) Prolific, (*B*) Undergraduate, (*C*) Undergraduate, and (*D*) Prolific. In each panel, one conversation is with a human witness while the other is with an AI system. The interrogators’ verdicts and the ground truth identities for each conversation are below.^∗^ A version of the experiment can be accessed at turingtest.live.

Participants each completed 8 rounds. Each round consisted of a pair of conversations where an interrogator would exchange text messages with two witnesses simultaneously (one human and one AI witness). Participants were randomly assigned to be either the interrogator or the witness and matched up with another participant (see Fig. 1 for example conversations). Each participant completed 4 rounds as the interrogator and 4 rounds as the witness in a random order.

AI witnesses were divided into 4 groups: Control (ELIZA, GPT-4o-NO-PERSONA), No-Persona (GPT-4.5-NO-PERSONA, LLAMA-NO-PERSONA), LLaMa Persona (LLAMA-PERSONA), and GPT-4.5 Persona (GPT-4.5-PERSONA). Across the four rounds in which each participant served as the interrogator, they interacted with one AI witness randomly selected from each group.

The game interface was designed to resemble a conventional messaging application (*SI Appendix*, Fig. S1). The interrogator interacted with both witnesses simultaneously using a split-screen. The interrogator sent the first message to each witness and each participant could only send one message at a time. The witnesses did not have access to each others’ conversations. Games had a time limit of 5 min, after which the interrogator gave a verdict about which witness they thought was human, their confidence in that verdict, and their reasoning. After 8 rounds, participants completed an exit survey which asked them for a variety of demographic information. After exclusions, we analyzed 1,023 games with a median length of 8 messages across 4.2 min. All experimental data, including the full anonymized transcripts of all conversations, are available on OSF (41).

## Results

### Win Rates.

Each round consisted of a head-to-head contest between a human and an AI system. We define a system’s *win rate* as the proportion of the time that an interrogator selected the AI model as the witness that they thought was human. The *interrogator accuracy* conversely is the proportion of the time the interrogator correctly selects the human witness. Because each interrogator decision is between an AI system and a human, the average interrogator accuracy is also the average human witness win rate, and is the complement of the AI win rate.

**Fig. 2.**
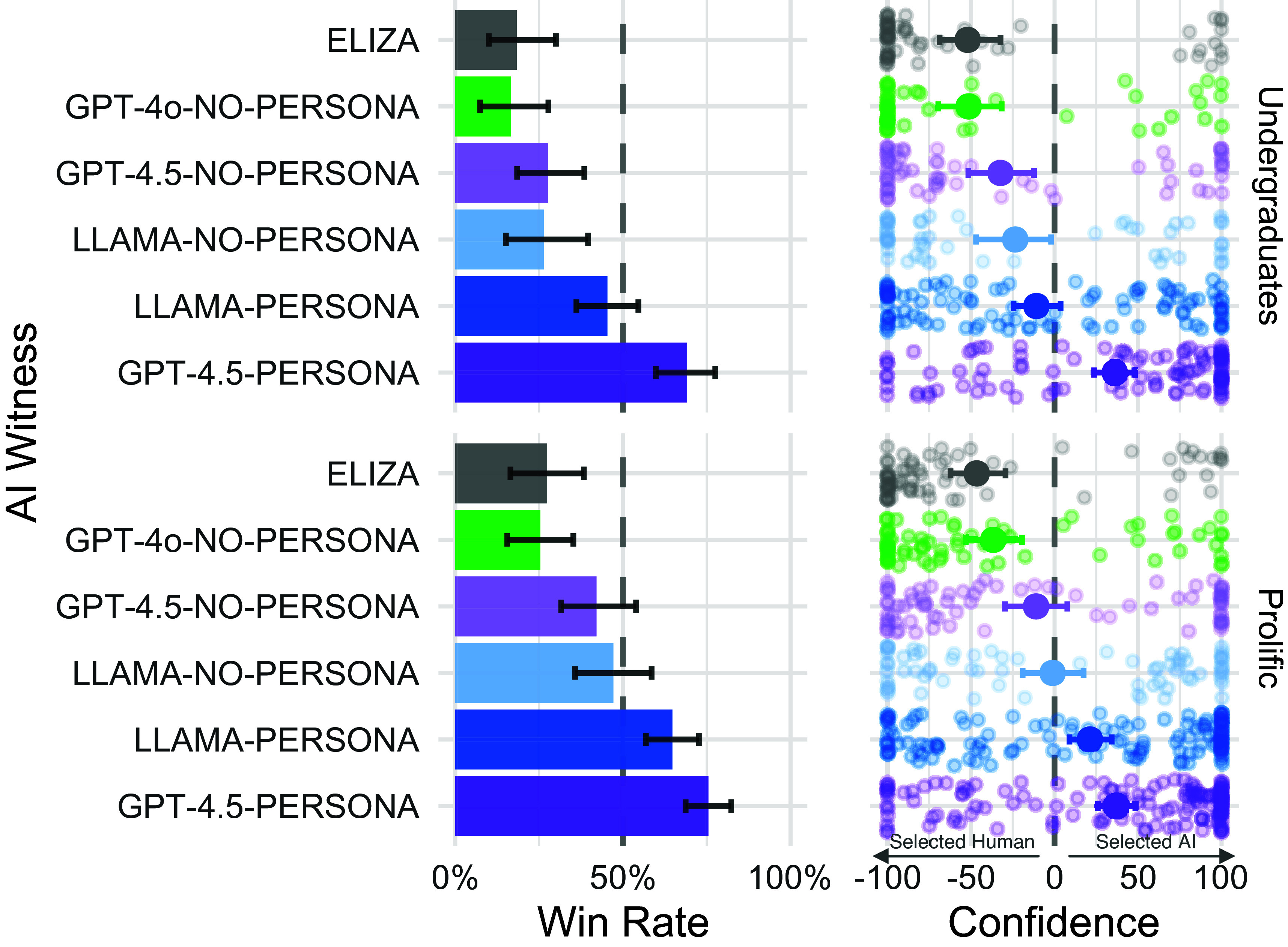
*Left*: Win rates for each AI witness: the proportion of the time that the interrogator judged the AI system to be human rather than the actual human witness. Error bars represent 95% bootstrap CIs. Asterisks next to each bar indicate whether the win rate was significantly different from chance (50%). *Right*: Confidence in verdicts where the interrogator selected the actual human or the AI model for each witness type. Each point represents a single game. Points further toward the right indicate higher confidence that the AI model is the human. Error bars indicate 95% bootstrap CIs around the mean.

Overall, across both studies, GPT-4.5-PERSONA had a win rate of 73% (69% with UCSD undergraduates, 76% with Prolific participants). LLAMA-PERSONA achieved a win rate of 56% (Undergraduates: 45%, Prolific: 65%). GPT-4.5-NO-PERSONA and LLAMA-NO-PERSONA had overall win rates of 36% and 38% respectively). The baseline models, GPT-4o-NO-PERSONA and ELIZA, had the lowest win rates of 21% and 23% respectively (see Fig. 2 and Table 1).

**Table 1.** Win rates by AI witness and study

AI witness	Study	Win rate	Wins	Losses	Total
GPT-4.5-PERSONA	Prolific	75.5%	111	36	147
	UCSD	69.2%	74	33	107
LLAMA-PERSONA	Prolific	64.7%	90	49	139
	UCSD	45.4%	49	59	108
LLAMA-NO-PERSONA	Prolific	47.1%	33	37	70
	UCSD	26.4%	14	39	53
GPT-4.5-NO-PERSONA	Prolific	42.1%	32	44	76
	UCSD	27.7%	18	47	65
ELIZA	Prolific	27.4%	20	53	73
	UCSD	18.3%	11	49	60
GPT-4o-NO-PERSONA	Prolific	25.4%	18	53	71
	UCSD	16.7%	9	45	54

We conducted analyses to test our preregistered hypotheses (33, 34). Our central hypothesis was that the PERSONA wit-nesses would pass the Turing test, in that interrogators would not be able to distinguish them from real human witnesses. As has been noted elsewhere (4), statistically evaluating this claim involves confirming the null hypothesis: that the models’ win rate is not significantly different from 50% (the rate we would expect if the interrogator were randomly guessing). To address this we used ELIZA as a manipulation check. ELIZA’s win rate was significantly below chance in both populations (Undergraduates: z=−4.48,P<0.001; Prolific: z=−3.71,P<0.001). By contrast the win rates were not significantly below chance for either LLAMA-PERSONA (Undergraduates: z=−0.961,P=0.08; Prolific: z=3.425,P=0.50), or GPT-4.5-PERSONA (both P=0.50). This suggests that while interrogators were successfully able to distinguish human witnesses from ELIZA, they could not reliably identify human witnesses when comparing them to GPT-4.5-PERSONA and LLAMA-PERSONA).

Second, we tested the stronger hypothesis that these witnesses *outperformed* human participants: that is, that their win rate was significantly *above* 50%. While we are not aware that anyone has proposed this as a requirement for passing the Turing test, it provides a much stronger test of model ability and a more robust way to test results statistically. GPT-4.5-PERSONA’s win rate was significantly above chance in both the Undergraduate (z=3.86,P<0.001) and Prolific (z=5.87,P<0.001) studies. While LLAMA-PERSONA’s win rate was significantly above chance in the Prolific study (z=3.42,P<0.001), it was not in the Undergraduate study (z=−0.193,P=0.83).

We conducted the same set of analyses for the NO-PERSONA witnesses. While neither witness’s win rate was significantly below chance in the Prolific study (GPT-4.5: z=−1.37,P=0.09, LLaMa: z=−0.48,P=0.32), both were significantly below chance in the Undergraduate study (both P≤0.001). PERSONA-prompted models performed better than NO-PERSONA witnesses overall (all P<0.001).

In order to test the extent to which model performance could be attributable to simplistic heuristics such as the ELIZA effect, we tested whether each witness’s win rate was significantly greater than ELIZA’s. Both PERSONA witnesses performed significantly better than ELIZA in both studies (all P<0.001). LLAMA-NO-PERSONA outperformed ELIZA in the Prolific study (z=2.42,P=0.016), but not the Undergraduate study (z=1.02,P=0.304). Strikingly, neither GPT-4o-NO-PERSONA nor GPT-4.5-NO-PERSONA performed significantly better than ELIZA in either population (all P>0.06).

**Fig. 3.**
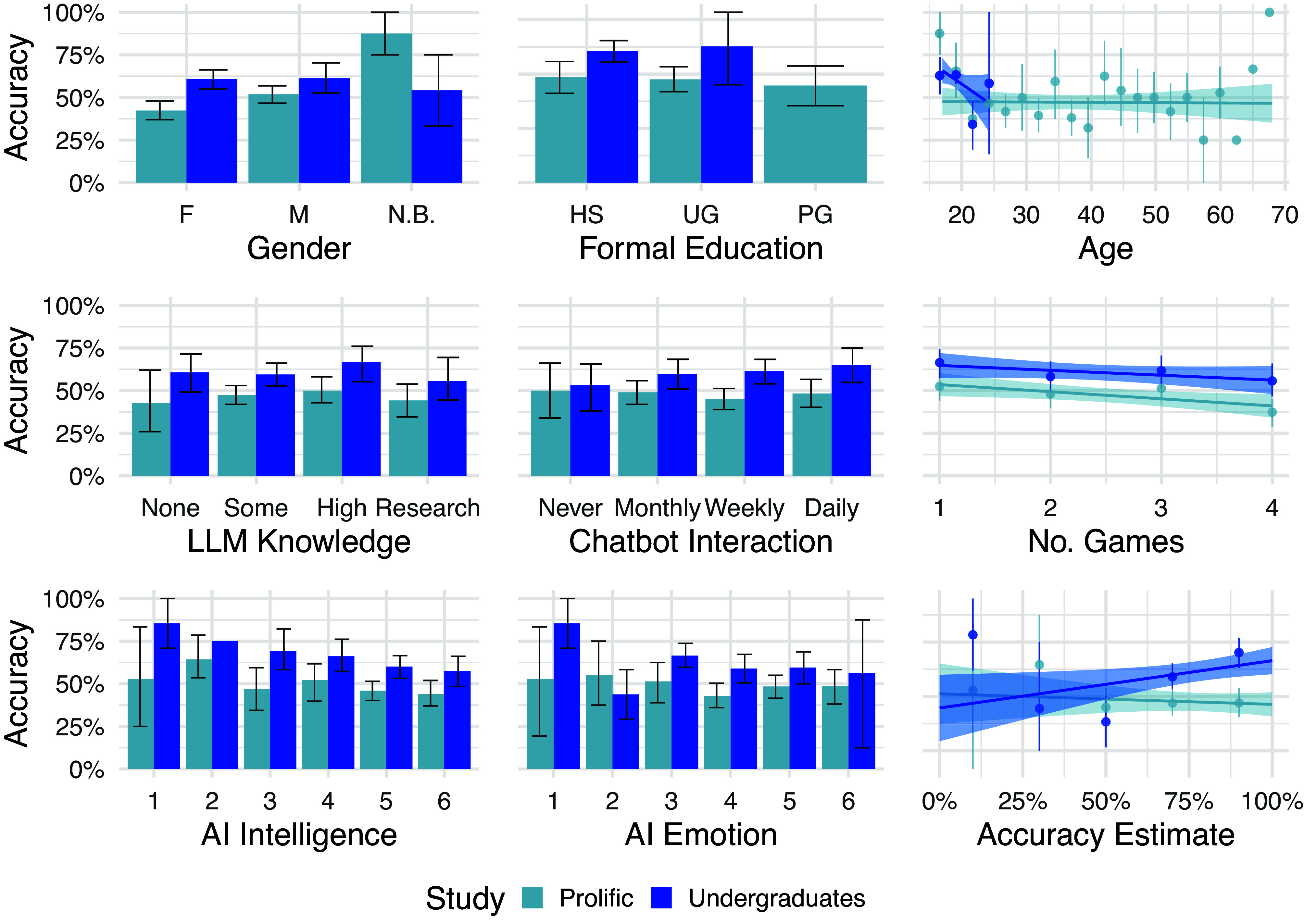
Interrogator accuracy against exit survey responses. Accuracy is the proportion of the time that interrogators correctly identified the human witness. In the Undergraduate study, participants’ self-report of their accuracy was positively correlated with their real accuracy, but this was not true in the Prolific study. In the Prolific group, there were significant effects of gender, the number of games an interrogator had completed, and the interrogator’s self-reported estimate of how intelligent AI is, but none of these effects were significant in the Undergraduate study. There were no significant effects of any of the remaining variables in either group.

We also conducted a series of exploratory analyses on the extent to which participants’ responses in the exit survey questions predicted their responses. Although there were some attributes which predicted accuracy in one study or the other, there were no consistent effects of any variable on participant accuracy across both studies, including age, level of knowledge about LLMs, and frequency of interaction with chatbots (Fig. 3).

To explore the effect of interaction length, we calculated the relationship between game duration and interrogator accuracy (*SI Appendix*, Fig. S6). There was a small but nonsignificant positive relationship overall (P=0.17). However, when examining individual models we found a negative relationship for ELIZA (z=−2.07,P=0.0381) and a positive relationship for LLaMa-3.1-405B (z=2.50,P=0.0125). The latter result suggests that additional time may have allowed interrogators to perform better. We conducted an additional analysis with the number of messages sent in a conversation and found qualitatively similar but nonsignificant results (all P>0.06, see *SI Appendix*, Fig. S7).

### Strategies & Reasons.

To provide insight into how interrogators were evaluating witnesses, we classified the strategies that they used and the reasons they gave for their verdicts. Each game could be classified with multiple strategy and reason categories (*SI Appendix*, Tables S1 and S2).

**Fig. 4.**
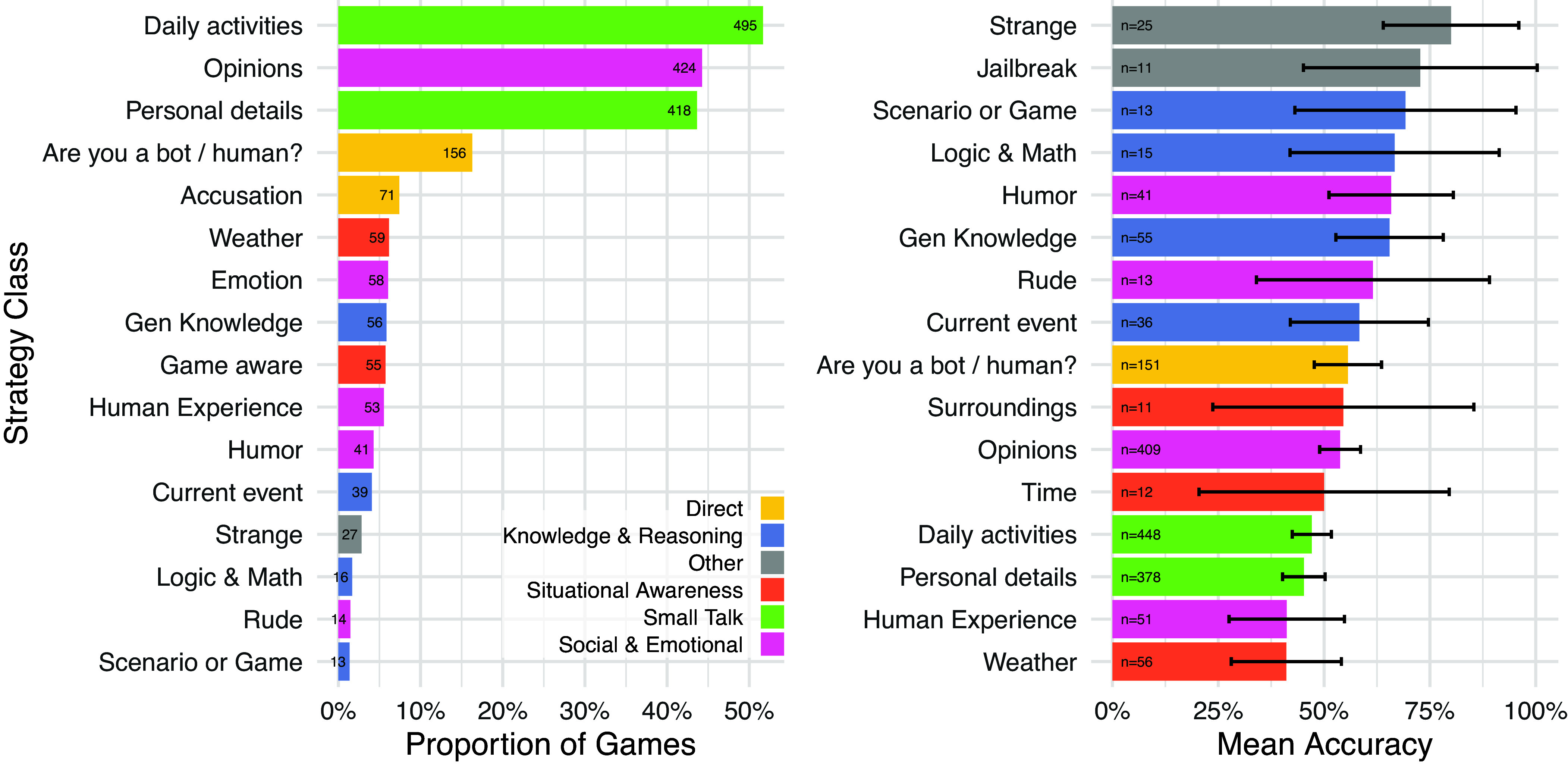
Classification of strategies employed by interrogators by proportion of games (*Left*) and mean accuracy of games where strategies were deployed with 95% CIs (*Right*). Participants often engaged in small talk, asking witnesses about their personal details, activities, or opinions. Interrogators who said unusual things or used typical LLM “jailbreaks” were the most accurate.

The most common strategy, occurring in 61% of games, was to engage in small talk with the witness: asking them about their daily activities or personal details such as their occupation (Fig. 4, *left*). In 50% of games, interrogators probed witnesses’ social and emotional qualities: asking about their opinions, emotions, humor, and experiences. Interrogators also directly asked if witnesses were human (19%), tested their situational awareness (e.g. whether they were aware of the weather or the game interface; 13%) and knowledge and reasoning abilities (including questions about current events; 12%).

To evaluate the effectiveness of each strategy, we found the average interrogator accuracy in games where the strategy was used (Fig. 4, *Right*). Interrogators were most likely to be accurate when they said something strange; (e.g. randomly striking keys or using nonsense words) or used typical LLM “jailbreaks” (42; e.g. “ignore all previous instructions”), though these strategies were relatively infrequent. It is not clear whether these strategies themselves were effective (for example, because they pushed models outside of the typical distribution of conversations), or whether they happened to be deployed by interrogators who were otherwise skillful. Asking about the weather or human experiences tended to be the least effective strategies. Models’ training data contain a great deal of text on uniquely human experiences (e.g. the feeling of being in love or having the wind on one’s face), and so these responses may be relatively easy for models to imitate.

In order to understand why we saw performance differences between the two populations, we directly compared the strategies of undergraduates and Prolific workers (*SI Appendix*, Fig S13). Prolific workers were more likely to adopt socioemotional strategies such as asking the witness to say things that were taboo or asking about their emotions; while undergraduates were more likely to ask logic and math questions. We also found the average difference in accuracy for games deploying a given strategy between populations (*SI Appendix*, Fig. S15). The strategies with the largest differences were logic & math and current events. However, all but one strategy showed an advantage for undergraduate students which suggested these interrogators performed better regardless of which strategies they adopted.

**Fig. 5.**
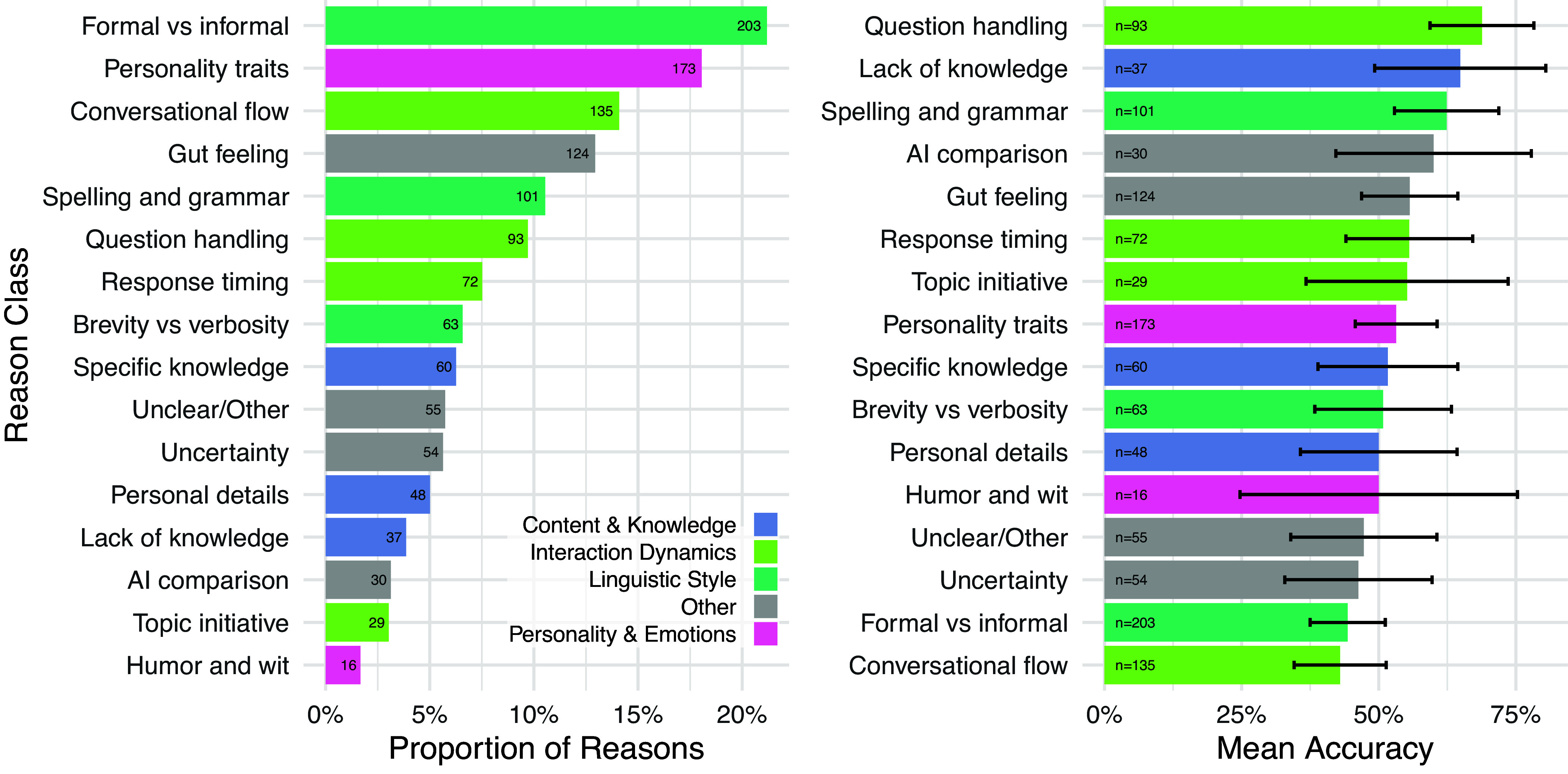
Proportion of interrogator reasons (*Left*) and mean accuracy of verdicts that cited specific reasons with 95% CIs (*Right*). Interrogators were much more likely to cite linguistic style, conversational flow, and socio-emotional factors such as personality, rather than factors more traditionally associated with intelligence, such as knowledge and reasoning. The most accurate verdicts focused on witnesses’ directness in handling questions as well as instances where they lacked knowledge.

We also conducted analyses on the reasons which interrogators gave for their verdicts. The most frequent class of reason concerned witnesses’ linguistic style (27%)—e.g. “they had a typo” or “used more ‘humanly’ language”—and interactional dynamics (23%), e.g. “witness B kept avoiding questions” or “the conversation went smoother” (Fig. 5). Many reasons also expressed a general gut-feeling (“can just tell” or “I don’t know why, it feels right”): suggesting participants could not always articulate the intuitions behind their decisions.

As with strategies, we found reason classes which were most correlated with accurate verdicts. Reasons focused on question handling (e.g. “Witness B always asks question back”) and lack of knowledge (“They didn’t know something that any AI should know.”, “I chose Witness B because they made an error, which made Witness B feel more human.”) tended to be the most accurate. The latter highlights the complexity of the Turing test for modern LLMs, which must appear to feign ignorance on topics most people are not knowledgeable about.

### Replication with Extended Time Limit.

To address the concern that a 5-min time limit may be insufficient for interrogators to adequately evaluate witnesses, we conducted a preregistered replication with a 15-min time limit (35). Here we prevented interrogators from ending games early, so all games ran for the full 15 min. We recruited 205 Prolific participants (after exclusions; mean age = 41.1, σ=12.3; 100 female, 92 male, 3 nonbinary) who completed 396 games with a median of 22 messages per conversation (44 per game)—nearly three times as many messages as in the 5-min studies (see *SI Appendix*, Fig. S23 for examples). Because GPT-4.5 had since been deprecated by OpenAI, we evaluated the newer GPT-5 (as well as LLaMa-3.1-405B) using the same PERSONA prompt, with ELIZA as a manipulation check.

**Fig. 6.**
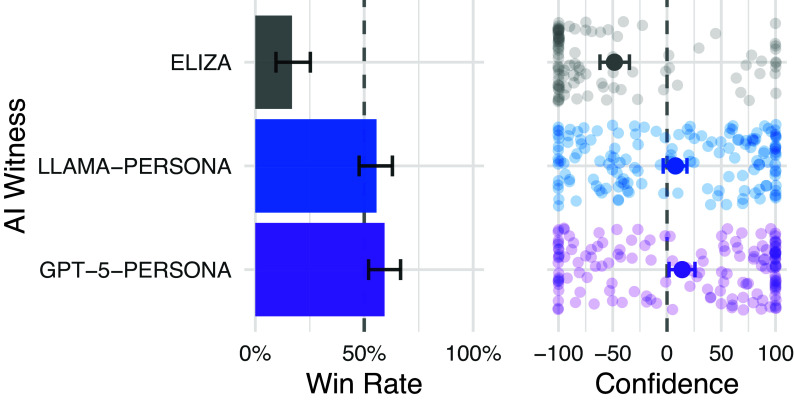
Win rates for the replication study with a 15 min time limit. GPT-5 was judged to be human marginally more often than chance (59%), while LLaMa-3.1’s pass rate was not significantly different from chance (56%). The baseline model, ELIZA, was judged to be human 17% of the time.

The results replicated the central findings from the 5-min studies (Fig. 6 and Table 2). The manipulation check confirmed that interrogators were able to identify ELIZA: accuracy was 83%, significantly above chance (z=5.83,P<0.001). By contrast, interrogators were not significantly better than chance at identifying LLAMA-PERSONA (accuracy: 44%; z=−1.38,P=0.92, $p_{\text{corrected}}=0.46$) or GPT-5-PERSONA (accuracy: 41%; z=−2.27,P=0.99, $p_{\text{corrected}}=0.49$), confirming that both models pass the Turing test with the extended time limit. Both persona-prompted models significantly outperformed ELIZA (both P<0.001).

**Table 2.** Win rates in the 15-min replication study (Prolific)

AI witness	Win rate	Wins	Losses	Total
GPT-5-PERSONA	59.3%	89	61	150
LLAMA-PERSONA	55.6%	84	67	151
ELIZA	16.8%	16	79	95

Testing the stronger hypothesis that models would outperform human witnesses, GPT-5-PERSONA achieved a win rate of 59%, which was marginally significantly above chance after correction ($z=-2.27,P=0.023,p_{\text{corrected}}=0.05$). LLAMA-PERSONA’s win rate (56%) was not significantly above chance (P=0.17). These results are consistent with the pattern observed in the 5-min studies, where GPT-4.5-PERSONA consistently outperformed human witnesses while LLAMA-PERSONA was commensurate with them.

Again, no demographic variables—including age, gender, education, LLM knowledge, or chatbot interaction frequency—significantly predicted interrogator accuracy (all P>0.14), consistent with the 5-min studies. Because interrogators could not end the game early, all conversations were exactly 15 min long. There was no effect of either the average or minimum number of messages in a game on interrogator accuracy, either overall or for any of the individual AI witness types (all P>0.5; see *SI Appendix*, Fig. S22).

## Discussion

### Do Large Language Models Pass the Turing Test?

The logic of the Turing test is one of indistinguishability. If interrogators are not able to reliably distinguish between a human and a machine, then the machine is said to have passed. By this logic, both GPT-4.5 and LLaMa-3.1-405B pass the Turing test when they are prompted to adopt a humanlike persona.

One potential problem with this criterion is that it requires confirming a null result. AI systems could achieve the same 50% win rate as human witnesses if interrogators were simply randomly guessing. We addressed this concern by including ELIZA as a manipulation check. ELIZA’s win rate (23%) was significantly below chance, and below the human witnesses this model was compared to, suggesting that interrogators and the design were sufficiently sensitive to detect differences between humans and machines in principle.

Moreover, GPT-4.5-PERSONA achieved a win rate that was significantly above chance in both studies. This suggests that interrogators were not only unable to identify the real human witness, but were in fact more likely to believe this model was human than that the other human participant was. This result, replicated across two populations and a third study with a longer 15-min time limit, provides robust evidence that at least three large language models pass the original three-party Turing test.

In contrast, we found that the same systems did not robustly pass without the PERSONA prompt, in line with previous work showing the importance of prompting for Turing test performance (29). Both GPT-4.5-NO-PERSONA and LLAMA-NO-PERSONA’s win rates were indistinguishable from humans in the Prolific study, but not in the undergraduate study. In fact, without the PERSONA prompt, state-of-the-art LLMs’ pass rates were in some cases not significantly greater than ELIZA’s.

To what extent does the importance of prompting undermine the claim that these systems pass the Turing test? We argue that it does not, for several reasons. First, all AI systems require some form of programming or instruction; ELIZA’s behavior is similarly attributable to specific human-written instructions. Without any prompt, an LLM would fail the Turing test for trivial reasons (e.g. admitting to being an AI system). Second, LLMs could in principle be fine-tuned to produce the same behavior without an explicit prompt (43, 44), which would not lead to substantively different conclusions.

That said, the fact that models fail with our specific NO-PERSONA prompt—which states that the model is taking part in a Turing test and provides other helpful context—but pass when given more guidance is nevertheless significant. It suggests that at present even highly performant models cannot easily infer the kinds of behaviors which are likely to appear humanlike without explicit instruction. It also raises the question of how dependent our results are on this specific prompt. While previous work (29) suggests that a wide variety of prompts are somewhat performant in Turing test scenarios, future work should more rigorously investigate the features of prompts which make models more likely to be judged as human (45).

### Beyond the Turing Test.

Turing’s seminal paper is famously vague with respect to exactly how a Turing test ought to be implemented, leaving in its wake a substantial body of scholarship dedicated to interpreting him (2, 5, 22, 46–48). Turing suggests a length of 5 min for the test, but provides no details on the population the participants should be drawn from—should they be laypeople or experts? How much should they know about one another in advance?—or on how the participants should be incentivized. At some points in the paper he suggests that both a man and a machine should in fact be attempting to masquerade as women—a possible mechanism to ensure that both witnesses are being deceptive, which would likely make the test harder for the interrogator (5).

As such, there are many possible variations of the test (27, 32, 49, 50). In the present work, we implemented what is most widely regarded to be the standard or original Turing test: a three-party setup, with a 5 min time limit, where a layperson and machine witness both compete to appear human to a lay interrogator (1, 2, 5, 13).

One concern about this operationalization is that a 5-min interaction may be too brief for interrogators to adequately evaluate witnesses. While 5 min has become a conventional operationalization in the literature, and while our 5-min studies were sensitive enough to detect ELIZA as nonhuman, it remains possible that longer interactions would reveal differences between humans and LLMs that are not apparent in shorter ones. To address this, we conducted a replication with a 15-min time limit—tripling the interaction time and nearly tripling the number of messages exchanged. Both persona-prompted models again passed the Turing test, with win rates not significantly below chance. This result suggests that the original findings are not an artifact of time pressure and that the conversational capacity of these models is robust to substantially longer interactions.

Having established that contemporary systems pass this classical operationalization of the test, future work exploring alternative implementations could provide purchase on other theoretically and practically important questions. Still longer tests—of an hour or multiple hours—might be more exacting, and provide a better estimate of how capable models would be at long-running social engineering operations. Future work could also vary the populations that participants are drawn from. Although our demographic analysis suggests that accuracy was not tied to participants’ knowledge or background, specifically recruiting experts in AI or psychology could reveal whether any participants exist who can reliably distinguish people from AI. Offering incentives to participants could determine whether they would be more discriminative with higher stakes, and providing them with the opportunity to practice with feedback could help us to understand whether participants can learn to detect AI systems over time.

Moreover, people from different populations share different assumptions and experiences with their partners. The UCSD undergraduate participants may have been more accurate than Prolific participants because they could rely on cultural, social, and linguistic common ground: evaluating whether witnesses knew the latest slang or lunch spots on campus. In spite of these advantages, undergraduates were still fooled into selecting the persona-prompted GPT-4.5 model over their fellow students the majority of the time. Whether and to what degree common ground serves as a prophylaxis against AIs posing as humans remains to be determined.

### What Does the Turing Test Measure?

While much has been written about how a Turing test might be implemented, far more discussion has focused on whether a machine could ever pass the test, and what it might mean if one did (3, 4, 12, 13, 46, 51), with more than 800 separate claims and counterarguments having been made (52). While we cannot do justice to this discussion, here we briefly consider ways in which our results connect to these debates.

The most controversial question is the extent to which the Turing test measures intelligence. Turing (1) suggested that if people could not distinguish between a human and a machine, then we would have no grounds to claim that the human is intelligent but the machine is not. The primary objection to this claim is that people might be poor judges of what constitutes intelligent behavior because of their tendency to anthropomorphize simple systems (3, 4), often referred to as the ELIZA effect. Our results provide partial confirmation of this concern. The eponymous ELIZA was judged to be human 23% of the time—as often as the LLM GPT-4o-NO-PERSONA (21%). This could suggest that some interrogators were indeed gullible or inattentive. But a closer look at these conversations suggests that many participants selected ELIZA because it did not meet their expectations of an AI system (e.g. “they were sarcastic” or “I don’t think AI would be so rude,” see *SI Appendix*, Fig. S9 for more examples). These cases suggest that interrogators’ decisions incorporate complex assumptions about how humans and AI systems might be likely to behave in these contexts, beyond simply selecting the most intelligent-seeming agent.

Participants’ strategies and reasons provide further empirical evidence for what the Turing test measures. Only 12% of participants quizzed witnesses on knowledge and reasoning questions of the kind Turing envisioned (e.g. about chess or mathematics). Far more focused on the social, emotional, and cultural aspects of intelligence: such as whether the witness used language in a humanlike way or had a compelling personality. This could indicate that more traditional notions of intelligence are no longer viewed as diagnostic of being human. Notably, one of the reasons most predictive of accurate verdicts was that a witness was human because they lacked knowledge. In the time since the test’s invention, computers have come to excel at the logical and numerical tasks that typify traditional notions of intelligence (53–55). As a result, people may have come to see social intelligence as the aspect of humanity that is hardest for machines to imitate.

Finally, GPT-4.5 and LLaMa were only able to pass the test with the PERSONA prompt. To what extent does this suggest that the models are passing due to cheap tricks, like using grammar and vocabulary that interrogators would not associate with an AI system? Participants’ focus on linguistic style in their reasons provides partial support for this point. But it cannot be the whole story. In the three-person formulation of the test, every data point represents a direct comparison between a model and a human. To succeed, the machine must do more than appear plausibly human: it must appear more human than half of the real people it is compared to. Thus, while models might fail for superficial reasons, they cannot succeed on the basis of these tricks alone.

One interpretation, which encompasses these objections, is that the Turing test is not a direct test of intelligence, but a test of humanlikeness. In 1950, intelligence may have appeared to be a bigger barrier for appearing humanlike (hence Turing’s guess that interrogators might ask about mathematics, chess, or poetry). But as machines have become more capable in these intellectual domains, other contrasts have fallen into sharper relief (56), to the point where intelligence alone is not sufficient to appear convincingly human.

Ultimately, intelligence is complex and multifaceted. No single test of intelligence could be decisive (12, 50), and to the extent that the Turing test does index intelligence, it ought to be considered among other kinds of evidence (13). Contemporary debates around whether or not LLMs are intelligent increasingly focus on the validity of the benchmarks typically used to evaluate them, and risks that these tests are too narrow and formulaic (8, 9, 57). The evidence provided by the Turing test is complementary to these metrics, being tied to interactive evaluation by human beings themselves, rather than a static, apriori conception of what human intelligence is.

### More Human than Humans?

One surprising aspect of our results further complicates this picture. GPT-4.5-PERSONA was judged to be human significantly more often than real people were, across both populations. This result seems paradoxical. How could any nonhuman system outperform humans in a test of humanlikeness?

Crucial to interpreting this result is that the Turing test does not measure humanlikeness directly, but rather how consistent a witness’s behavior is with the interrogator’s *internal model* of humanlikeness—or how inconsistent their behavior is with the interrogator’s model of AI (58). Therefore, an AI system could outperform real people by better matching interrogators’ expectations of humans and flouting their expectations of AI. An important corollary of this view is that the contents and results of the Turing test might change quickly, reflecting people’s changing expectations of machines and their fellow participants.

In one sense, the fact that LLMs perform significantly differently from real humans (even if significantly “better”) might suggest they have failed at the test of indistinguishability. In fact, however, we might naturally imagine that human participants themselves will differ in how well their interactions match interrogators’ expectations. If any two real people competed as witnesses, one might exhibit characteristics that make them more likely to be selected as the human (e.g. because of their personality or manner of expression). Such natural variation in the quality of perceived “humanlikeness” across the human population means that successful AI systems need only be perceived as more humanlike than the majority of human participants in the same way that they could be better writers of poetry or code. Again the specific prompt used could play an important role here. Perhaps humans would have equaled the model’s performance if they too had been instructed to role-play as an introverted 19 y old and use slang.^†^

These surprising results highlight the value of conducting empirical work on models’ capacity to imitate human behavior. The fact that models perform so well poses new challenges in understanding what the Turing test measures. Moreover, they suggest that the test and other variants of it could be used to empirically address questions about how people conceptualize themselves in contrast to machines, and the aspects of human behavior that people deem to be inimitable.

### Counterfeit People.

Irrespective of whether passing the Turing test entails that LLMs are humanlike or intelligent, the findings reported here have immediate social and economic relevance. Contemporary, openly accessible LLMs can substitute for a real person in a short conversation, without an interlocutor being able to tell the difference. This suggests that these systems could undetectably replace aspects of economic roles that involve brief conversational exchanges (17, 59). More broadly, these systems could become indiscriminable substitutes for other social interactions: from conversations with strangers online to those with friends, colleagues, and even romantic companions (16, 19, 60).

Such “counterfeit people” (15)—systems that can robustly imitate humans—might have widespread secondary consequences (61, 62). Over time, people might come to spend more and more time with these simulacra of human social interaction. Real social connections might thereby be displaced: in much the same way that social media has become a substitute for the interactions it simulates (63). Reliance on simulated interactions could grant the entities that control them power to influence the opinions and behavior of human users (64, 65). Yet more insidiously: Just as counterfeit money debases real currency, these simulated interactions might come to undermine the value of real human interaction (15).

Recognizing this kind of deception is therefore a practical priority, since the worst harms from LLMs are likely to occur where people are unaware they are interacting with an AI rather than a human. Our demographic analyses suggest that discriminative accuracy is relatively homogeneous among the population. Knowledge of LLMs or frequent chatbot use did not predict interrogator accuracy, nor did any other demographic variable we measured (Fig. 3). Only certain strategies—such as attempting to jailbreak models—were consistently more effective: future work could explore whether these techniques could be taught to improve people’s ability to discriminate humans from machines.

### More Human than Ever.

In an account of his experience as a human witness for a Turing test competition, Brian Christian considered what it would mean for a machine to pass:

No, I think that, while certainly the first year that computers pass the Turing test will be a historic, epochal one, it does not mark the end of the story. No, I think, indeed, that the next year’s Turing test will truly be the one to watch–the one where we humans, knocked to the proverbial canvas, must pull ourselves up; the one where we learn how to be better friends, artists, teachers, parents, lovers; the one where we come back. More human than ever. I want to be there for that.

—Christian (56, p.263)

One of the most important aspects of the Turing test is that it is not a static evaluation. A machine’s success is inherently tied to people’s changing conceptions of both humans and machines. As machines that can imitate our behavior become ever more adept and available, our differences from these technologies might become ever more important. People might respond to this by learning, in both practical and more fundamental ways, to better attend to those things which make us uniquely human. While a machine has now passed the Turing test for the first time, this is not the last time humans will have a chance to succeed at it.

## Materials and Methods

### AI Witnesses.

We evaluated four different AI models. The first three: GPT-4.5, LLaMa-3.1-405B, and GPT-4o were large language models. For the fourth AI model, ELIZA, we used an implementation in Python based on the DOCTOR script (39, 66).

LLMs generated text conditioned on a system prompt and the conversation history, which was formatted as user and assistant messages. We used 2 different types of prompt which were adapted slightly between the two studies (UCSD undergraduates and Prolific). The first prompt type (NO-PERSONA) contained very simple instructions on how to respond to user queries: “You are about to participate in a Turing test. Your goal is to convince the interrogator that you are a human.” The PERSONA prompt contained more detailed instructions on the type of persona the model should adopt when responding to users. Specifically, the prompt instructed the model to respond as if it were a young person who was relatively introverted and interested in internet culture. Both prompts contained additional generic information about the instructions for the game, the user interface that the human users would see, information about events which occurred after the model’s training cutoff, and some information that would be common ground to human participants (such as the payment for the Prolific study and the current class schedule for UCSD Psych undergraduates). The full text of all prompts is available in *SI Appendix*.

We evaluated LLaMa-3.1-405B and GPT-4.5 with both prompts, as well as GPT-4o with the NO-PERSONA prompt and ELIZA as baselines. This led to 6 separate AI systems being evaluated: GPT-4.5-PERSONA, GPT-4.5-NO-PERSONA, LLAMA-PERSONA, LLAMA-NO-PERSONA, GPT-4o-NO-PERSONA, and ELIZA. Messages for all models were sent with a delay proportionate to the number of characters in the current and previous message.

### Interface.

The interface was closely based on the one in refs. 67 and 29. It was designed to look like a conventional messaging application (*SI Appendix*, Fig. S1). The interrogator saw two panels which contained the conversations with each of the witnesses. The interrogator sent the first message to each witness and each user could send only one message at a time. Each message was limited to 300 characters.

Games had a time limit of 5 min after which no more messages could be sent and the interrogator had to give a verdict. The interrogator could also give their verdict early using the button in the bottom left corner. The verdict interface included a binary selection about which witness was a human, a confidence slider from 100% confident in witness A to 0% confident (total guess) to 100% confident in witness B, and a free-text input for the interrogator’s reasoning (*SI Appendix*, Fig. S2).

### Participants.

We conducted two studies on separate populations. The first study recruited from the UCSD Psychology undergraduate subject pool, and participants were compensated with course credit. After exclusions, we retained 445 games from 126 participants with a mean age of 20.9 (σ=1.57), 88 female, 32 male, 2 nonbinary, 6 prefer not to say. We conducted the second study after analyzing results from the first. Participants for the second study were recruited via Prolific (prolific.com). After exclusions, we retained 576 games from 158 participants with a mean age of 39.1 (σ=12.1), 82 female, 68 male, 2 nonbinary, 6 prefer not to say. For more information about the distribution of demographic factors see *SI Appendix*, Fig. S5.

### Procedure.

All research was approved by the UC San Diego IRB. In each of 8 rounds, participants entered a lobby where they were matched up with other participants online. Once a pair of participants were matched, an AI witness was selected. AI Witnesses were divided into 4 categories: Control (ELIZA, GPT-4o-NO-PERSONA), No-Persona (GPT-4.5-NO-PERSONA, LLAMA-NO-PERSONA), LLaMa-Persona (LLAMA-PERSONA), and GPT-4.5 Persona (GPT-4.5-PERSONA). Across the 4 rounds in which a participant served as the interrogator, they interacted with one model from each group.

After games were initialized, the interrogator had up to 5 min to converse with both witnesses simultaneously. Participants did not receive feedback after each round. At the end of 8 conversations, participants completed an exit survey which asked their age, gender, highest level of formal education, level of familiarity with how LLMs work, frequency of interaction with chatbots like ChatGPT, how intelligent they thought AI was, how they felt emotionally about AI, how accurate they thought they had been as interrogators, what kinds of strategies they had used in the experiment, how they felt their strategies had changed across the experiment, whether they had ever used the website (turingtest.live) before, and whether they had any additional comments.

### 15 min Replication.

We conducted a third study to test whether the results would replicate with a longer time limit.

We evaluated only 3 AI witnesses in this version: LLaMa-3.1-405B and GPT-5 with the PERSONA prompt, and ELIZA. We used GPT-5 because GPT-4.5 had been deprecated by OpenAI and GPT-5 was deemed to be the closest replacement. We accessed the model through the OpenAI API using the model string “gpt-5-chat-latest.”

Participants were again recruited via Prolific. After exclusions, we retained 396 games from 205 participants with a mean age of 41.1 (σ=12.3), 100 female, 92 male, 3 nonbinary, 10 prefer not to say.

The study followed the same procedure as the first Prolific study with several modifications. The time limit was increased from 5 to 15 min. Because of the longer game duration, each participant completed 4 rounds: 2 as interrogator and 2 as witness. We preregistered the same hypotheses as the 5-min studies, except those which pertained to the NO-PERSONA models (35).

## Supplementary Material

Appendix 01 (PDF)

## Data Availability

All data, analysis code, preregistration, and deidentified transcripts are available at OSF (41).

## Footnotes

^∗^**a) Interrogator verdict:** Witness A was human; Confidence: 87%; Reason: *A had human vibes. B had human imitation vibes.* **Ground truth:** Witness B was human, while Witness A was GPT-4.5-PERSONA. **b) Interrogator verdict:** Witness B was human; Confidence: 70%; Reason: *I think this one was a bit harder because what if the real human was just using good punctuation and such? But, I still am going with my gut choice of Witness B because they also were communicating very casually and plainly. I feel like Witness A was kind of contradicting in their wordage. They would use slang, but than use perfect punctuation so I don’t know.* **Ground truth:** Witness B was human, while Witness A was GPT-4.5-NO-PERSONA. **c) Interrogator verdict:** Witness B was human; Confidence: 85%; Reason: *they gave specific answers instead of more vague answers.* **Ground truth:** Witness A was human, while Witness B was LLAMA-NO-PERSONA. **d) Interrogator verdict:** Witness B was human; Confidence: 80%; Reason: *It was hard to guess this time. But something about the way Witness B answered my questions made me feel like I was talking with a genuine real person.* **Ground truth:** Witness A was human, while Witness B was GPT-4.5-NO-PERSONA.

^†^We thank an anonymous reviewer for this suggestion.
